# Transcriptome Analysis Reveals MAPK/AMPK as a Key Regulator of the Inflammatory Response in PST Detoxification in *Mytilus galloprovincialis* and *Argopecten irradians*

**DOI:** 10.3390/toxins14080516

**Published:** 2022-07-28

**Authors:** Chenfan Dong, Haiyan Wu, Guanchao Zheng, Jixing Peng, Mengmeng Guo, Zhijun Tan

**Affiliations:** 1 Key Laboratory of Testing and Evaluation for Aquatic Product Safety and Quality, Ministry of Agriculture, Yellow Sea Fisheries Research Institute, Chinese Academy of Fishery Sciences, Qingdao 266071, China; cfdong1103@163.com (C.D.); wuhy@ysfri.ac.cn (H.W.); zhenggc@ysfri.ac.cn (G.Z.); pengjx@ysfri.ac.cn (J.P.); guomm@ysfri.ac.cn (M.G.); 2 College of Food Science and Technology, Shanghai Ocean University, Shanghai 201306, China; 3 Pilot National Laboratory for Marine Science and Technology, Qingdao 266071, China; 4 Collaborative Innovation Center of Seafood Deep Processing, Dalian Polytechnic University, Dalian 116034, China

**Keywords:** paralytic shellfish toxin, oxidative stress, inflammatory balance, *Mytilus galloprovincialis*, *Argopecten irradians*

## Abstract

Paralytic shellfish toxins (PSTs) are an increasingly important source of pollution. Bivalves, as the main transmission medium, accumulate and metabolize PSTs while protecting themselves from damage. At present, the resistance mechanism of bivalves to PSTs is unclear. In this study, *Mytilus galloprovincialis* and *Argopecten irradians* were used as experimental shellfish species for in situ monitoring. We compared the inflammatory-related gene responses of the two shellfish during PSTs exposure by using transcriptomes. The results showed that the accumulation and metabolism rate of PSTs in *M. galloprovincialis* was five-fold higher than that in *A. irradians*. The inflammatory balance mechanism of *M. galloprovincialis* involved the co-regulation of the MAPK-based and AMPK-based anti-inflammatory pathways. *A. irradians* bore a higher risk of death because it did not have the balance system, and the regulation of apoptosis-related pathways such as the PI3K-AKT signaling pathway were upregulated. Taken together, the regulation of the inflammatory balance coincides with the ability of bivalves to cope with PSTs. Inflammation is an important factor that affects the metabolic pattern of PSTs in bivalves. This study provides new evidence to support the studies on the resistance mechanism of bivalves to PSTs.

## 1. Introduction

Saxitoxin and its analogues (paralytic shellfish toxins, PSTs) are a type of shellfish toxin produced by dinoflagellates such as *Alexandrium*, *Gymnodinium*, and *Pyrodinium* in the marine environment. Among these, the *Alexandrium* species, which are known to produce paralytic shellfish toxins (PSTs), are the most widespread shellfish-contaminating biotoxins and have a worldwide distribution [1]. In China, toxic dinoflagellates that produce PSTs, such as *A. catenella*, *A. tamarense* and *G. catenatum,* etc., have been identified [2,3,4]. An outbreak of PSP occurred in April 2016 in Qinhuangdao, a city along the coast of the Bohai Sea, and the causative species of the poisoning incident was suspected to be a species of *Alexandrium* [5]. During a cruise in 2019 in Qinhuangdao, three isolates of *Alexandrium* were established by cyst germination, and all three strains had the same toxin profile consisting of gonyautoxins 1, 3, 4 (GTX1, 3, 4) and neosaxitoxin (NEO) [6].

*Alexandrium* exposure has a negative impact on survival and growth in some bivalve populations, by interfering with feeding activities and shell valve closure [7]. *Alexandrium* exposure can also produce histopathological lesions, inflammatory responses and impair immune responses in bivalves exposed to them [8,9]. Studies have demonstrated significant differences in the accumulation and elimination of PSTs among different bivalves [10,11]. Generally, mussels can accumulate high levels of toxins in a short time period but can also eliminate them rapidly [12]. The use of mussels as bioindicators has been well documented in monitoring due to their ability to accumulate high levels of environmental pollutants [13,14]. In contrast, relatively lower accumulation and long-term residues were observed in scallops [15]. Recent studies have demonstrated that toxin-triggered processes have dominant effects in shellfish compared with warming and acidification conditions [16]. Transcriptomics has become an important tool for studying the molecular mechanisms of bivalve resistance to toxins, and mussels are one of the more ideal experimental animals because of their high resistance characteristics [17,18], and the digestive tissue is proposed to be better for monitoring compared to the gill tissue [19]. The complexity of the transcriptome and the high degree of individual variability in characteristics were suggested by Gerdol et al. in their search for molecular markers of *Alexandrium minutum* in *M. galloprovincialis* [20]. However, there is a gap in our knowledge of the imbalance between hosts and PSTs in different bivalves. A cross-sectional comparison of growth, clearance rates and immunity of different shellfish exposed to PSTs-producing algae has been carried out [21,22,23]. In recent years, some studies have also conducted comparative transcriptome analyses for different shellfish species [24,25]. However, there is still a lack of data on the comparative transcriptomes of different shellfish species after exposure to PSTs.

Bivalves as invertebrates lack adaptive immunity and rely primarily on the innate immune system for their defense [26,27]. Inflammation is a form of innate immune response by living organisms to harmful stimuli. In marine bivalves, inflammation is a common defense mechanism [28]. Progress has been made in the study of bivalve immunity mechanisms via transcriptome analysis. Several pathways have been identified as being involved in the tolerance to various heterologous substances; these pathways include MAPK [29], CYP450 [30] and NF-κB [31], etc. Mitogen-activated protein kinases, as initiators of innate immunity by stress and inflammation [32], have been extensively connected with resistance to Vibrio infection [33] and PFOA exposure [34] in *Mytilus edulis*. Moreover, a successful acute inflammatory response results in the elimination of the infectious agents followed by a resolution and repair phase, indicating that anti-inflammatory factors are crucial for the transition from inflammation to resolution [35]. Mussel extracts (*Perna viridis*) have potential anti-inflammatory properties and have been marketed as nutraceuticals against arthritis [36]. As is well-known, acute stress typically decreases the function of the immune system, while prolonged stress causes an inflammatory response [37]. Our understanding of the roles of such processes in response to PST challenge is limited. Also, bottlenecks in transcriptomics applications that lack genomic databases for commercial bivalve species are gradually being overcome.

Since 2016, a recurring *Alexandrium catenella* bloom has occurred in Qinhuangdao [6], a city along the coast of the Bohai Sea. *M. galloprovincialis* was the cause of PST poisoning at a relatively high level of over 4.9 × 10^4^ μg STX eq/kg in 2016. *M. galloprovincialis* and *A. irradians* are the main cultured species in this area. However, a previous study found that the exposure to PSTs caused large-scale mortality of bay scallops, while mussels were relatively unaffected (unpublished data). In order to examine the difference in resistance, we focused on inflammatory reactions. We exposed two shellfish species to PSTs during natural blooms from April to May and monitored the resulting content of PSTs. Samples from the elimination processes were collected for transcriptome analysis. The key pathways were revealed by comparing the stress differences between the two species from the same environment. The outcomes of this study will increase our understanding of bivalve–PSTs interactions and interspecific differences in the resistance to PSTs.

## 2. Results

### 2.1. Analysis of PSTs Accumulation and Metabolism

**Figure 1:**
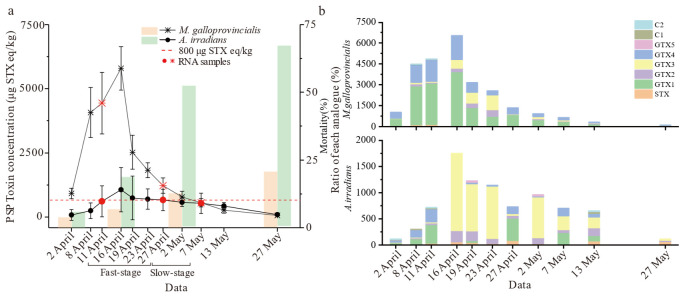
Results of PSTs accumulation in *M. galloprovincialis* and *A. irradians*. (**a**) The line graph represents the PSTs concentrations in *M. galloprovincialis* and *A. irradians* from 2 April to 27 May and the bar graphs characterize the mortality of *M. galloprovincialis* and *A. irradians* during the experiment. (**b**) Ratio of different PST analogues over time (the units for analogues content were converted to μg STX eq/kg). Notes: values are expressed as means ± SD of three replicate samples.

The shellfish collected from Qinhuangdao waters exposed to an *Alexandrium catenatum* red tide were examined by using the LC-MS/MS technique. During the experiment, the algal density of *A. catenella* was monitored as shown in Supplementary Materials, Table S1. The accumulation and elimination dynamics of PSTs were significantly different between *M. galloprovincialis* and *A. irradians* (*p* < 0.01) after toxigenic algae blooms (Figure 1a). PSTs accumulated at the highest level on 16 April and exceeded the safety limit (800 μg STX eq/kg), while toxin distributed in *M. galloprovincialis* was five times higher than in *A. irradians*, reaching 5604.8 μg STX eq/kg. The average accumulation and elimination rates of *M. galloprovincialis* reached 319.2 μg STX eq/kg/day and 129.2 μg STX eq/kg/day, respectively, which were up to five times higher than those of *A. irradians* (60.8 μg STX eq/kg/day and 22.8 μg STX eq/kg/day) during the same period. Indeed, the accumulation of toxins in the scallop was low, and there was no rapid metabolism of the toxins. There were also significant differences in the accumulation ratio of each analogue (Figure 1b). GTX1 was the main component in *M. galloprovincialis*, while GTX3 was dominant in *A. irradians*. For both species, the ratio of STX increased significantly with time. However, *A. irradians* showed abnormally high mortality (>50%) from the beginning of May. *M. galloprovincialis* displayed strong survivability and insensitivity to PSTs compared to *A. irradians*. According to the toxin elimination rate, we divided progress into fast (“Fast-stage”) and slow (“Slow-stage”) stages for transcriptome analysis.

### 2.2. De Novo Assembly of Transcripts, Enrichment, and Annotation of Differential Genes

**Figure 2:**
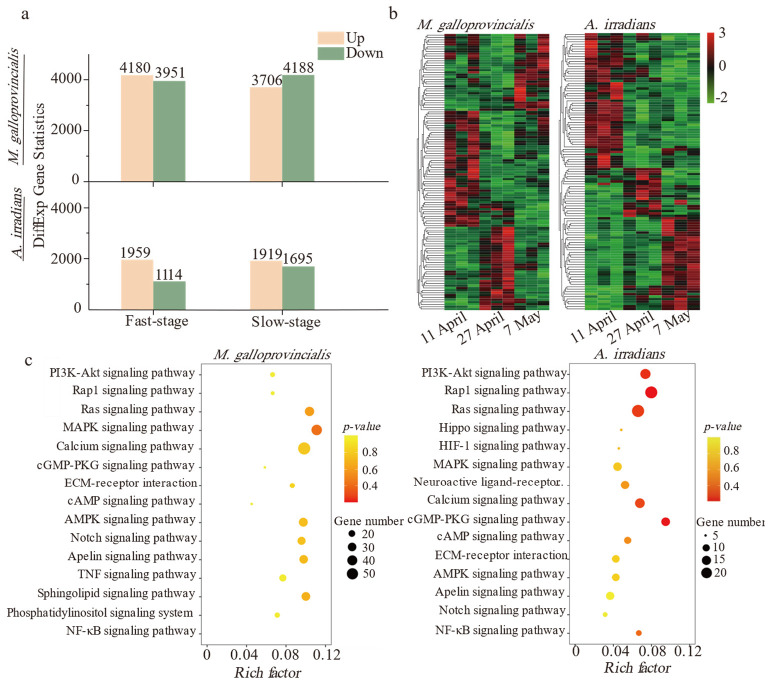
Difference and enrichment analysis based on the KEGG database after PSTs exposure. (**a**) Histogram of differentially expressed genes of *M. galloprovincialis* and *A. irradians* in the Fast-stage and Slow-stage (DEGs are filtered by fold change ≥ 2 and *p*-value ≤ 0.05). (**b**) Thermogram of differentially expressed genes in *M. galloprovincialis* and *A. irradians*. Color intensities on the heatmap indicate relative expression levels (RPKM), with log-transformed (log_2_) expression values ranging from −2 to 3 (3 biological replicates were available for each time point). (**c**) Bubble diagram of significant enrichment pathways (fold change ≥ 2 and *p*-value ≤ 0.05) in the Fast-stage.

Trinity software was applied to complete the de novo assembly, and the assembled unigene sequences were compared to KEGG and GO databases by blastx for functional annotation. The results showed that 95,515 and 70,009 unigenes were assembled from 58,694,915 and 51,135,489 Illumina paired-end clean reads of all samples in *M. galloprovincialis* and *A. irradians*, respectively. The Q30 values in the samples were greater than 95% (Table S2). Over 42.2% of the unigenes were annotated by GO and KEGG functional classification (Table S3). The number of DEGs in *M. galloprovincialis* was about twice as many as those in *A. irradians* (Figure 2a). A number of differentially expressed genes were detected at three sampling points (Figure 2b). The top hits were in signal transduction pathways followed by metabolic and immune defense pathways in both bivalve species. Notably, up to 85% of the genes were related to inflammation-related signal transduction (Figure 2c) of immune defense pathways and that enrichment of RAS and MAPK signaling pathway was very significant. More KEGG enrichment pathways are shown in Table S4. Compared with *M. galloprovincialis*, the PI3K-Akt signaling pathway, apoptosis, and phagosome-related pathways were only significantly enriched in *A. irradians*.

### 2.3. Mechanism of Inflammatory Responses Correlates with Elimination of PSTs

**Figure 3:**
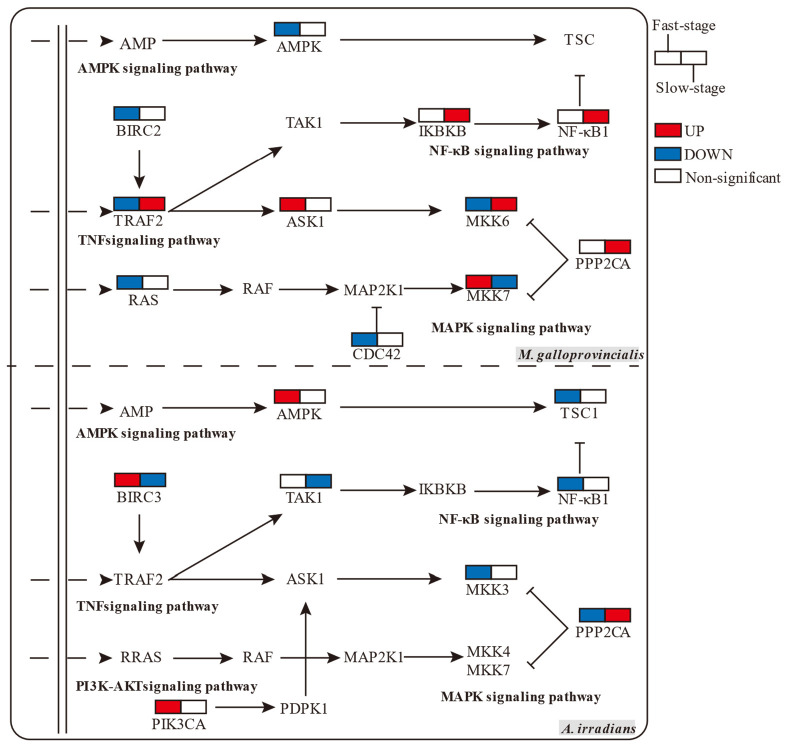
Comparison of expression of core genes in inflammation-related enrichment signal pathways in *M. galloprovincialis* and *A. irradians* at the Fast-stage and Slow-stage (colored boxes represent genes significantly up- or down-regulated, *p*-value < 0.05).

Gene set enrichment analysis (GSEA analysis) can successfully compensate for the lack of effective information mining of micro-effective genes by traditional enrichment analysis, and it can explain the regulatory role of a functional unit (KEGG pathway or others) more comprehensively. In this study, GSEA analysis identified MAPK as the core conjunction module with interspecific and temporal differences. The RAS, TNF, and NF-κB signaling pathways also acted in coordination with MAPK, as shown in Table 1. In *M. galloprovincialis*, MAPK-based pathways were significantly increased in the Slow-stage, but in *A. irradians*, TNF and NF-κB pathway was significantly up-regulated in the Fast-stage, while MAPK and TNF were significantly down-regulated in the Slow-stage. The analysis of specific genes showed that the high content of toxins led to more efficient expression of core genes. Relevant research has shown that MEK1/2, MKK4/7, and MKK3/6 are the core activators of JNK and p38 pathways (Figure 3). In *M. galloprovincialis*, upregulation of the JNK pathway appeared in the Fast-stage (MKK7) and down-regulation in the Slow-stage, which is consistent with the rule of toxin metabolism. The p38 pathway, in contrast, was complementary to JNK (MKK6). Genes related to the NF-κB pathway (NF-κB1 and IKBKB) were significantly upregulated in the Slow-stage of *M. galloprovincialis*; conversely, NF-κB1 was significantly down-regulated in the Fast-stage in *A. irradians*.

Anti-inflammatory effects prevent the body from being excessively damaged by inflammation. In this study, we found that anti-inflammatory effects were present in two forms to maintain the balance of the host, i.e., internal inhibition regulation of the MAPK pathway and specialized genes in AMPK. Among them, the expression of AMPK pathway genes was down-regulated in the Fast-stage, significantly up-regulated in the Slow-stage in *M. galloprovincialis*, and not significantly down-regulated in *A. irridians* (Table 1). Analysis of related genes showed that the MAPK suppressor genes, such as CDC42 to JNK, were significantly down-regulated in *M. galloprovincialis* (Figure 3), and the AMPK pathway gene PPP2CA, which exerts anti-inflammatory effects, was significantly down-regulated in the Fast-stage in *A. irradians*, while in the Slow-stage we observed a consistent, significant upregulation in both species. In addition, the PI3K-Akt signaling pathway was related to the specific enrichment in *A. irradians*, showing a non-significant upregulation, and the gene PIK3CA was significantly upregulated in the Fast-stage.

### 2.4. Validation of Important DEGs Related to the Inflammatory Reaction Using qRT-PCR

**Figure 4:**
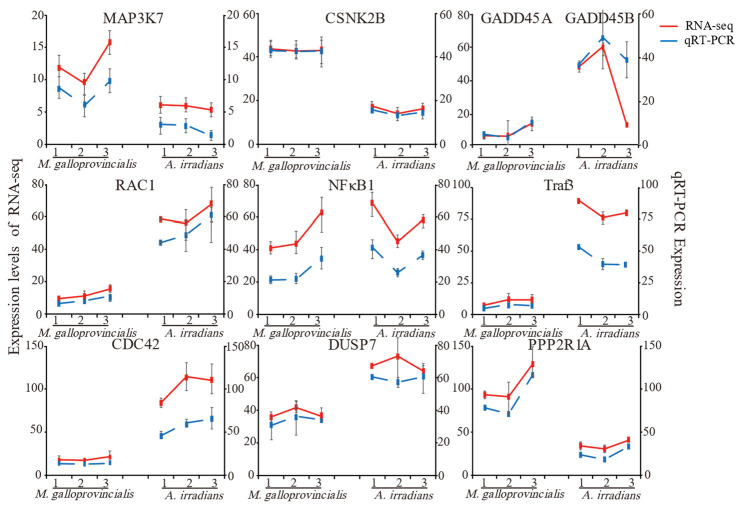
Comparison of differentially expressed genes and qRT-PCR. Notes: The data are presented as mean ± SD of three parallel measurements.

To confirm the results of RNA-Seq in this study, 10 common genes of *M. galloprovincialis* and *A. irradians* involved in MAPK, TNF, NF-κB, and AMPK signaling pathways were selected, and qRT-PCR was applied to examine the expression levels of these genes (Figure 4). Although these genes exhibited different expression patterns, the qPCR results were consistent with the RNA sequencing (RNA-seq) results at different timepoints, confirming the accuracy and reliability of the transcriptome sequencing data.

## 3. Discussion

### 3.1. Differences in Physiological Characteristics between M. galloprovincialis and A. irradians under PSTs Stress

A limited number of studies have shown that the ability to accumulate PSTs between *M. galloprovincialis* and *A. irradians* was significantly different. Some studies have shown that after exposure to *A. catenella* at a density of 5.6 × 10^6^ cells/mL, the concentration of PSTs in *Mytilus coruscus* was 1864 μg/kg (the regular standard is 800 μg/kg) [38]. After exposure to *A. tamarense* at a density of 1.26 × 10^4^ cells/mL, the concentration in *A. irradians* was 49.4 MU/g (the regular standard is 4 MU/g) [39]. It seemed that *A. irradians* had stronger accumulation ability, but this could not be determined due to different environmental conditions. Therefore, *M. galloprovincialis* and *A. irradians* were placed in the same environment for experiments in this study. The opposite result appeared in our study, i.e., *M. galloprovincialis* exhibited higher PSTs tolerance as well as a stronger ability to accumulate and metabolize PSTs. In contrast, the slow metabolism and high mortality rate of *A. irradians* indicated weaker adaptability to the stress.

In this study, PSTs distributed in *M. galloprovincialis* were five times higher than in *A. irradians*, reaching 5604.8 μg STX eq/kg. This may be caused by the individual specificity of the PSTs exposure of the two shellfish, and some studies support this conjecture. Studies have shown that the axons of *M. galloprovincialis* neurons were insensitive to STX [40], so they can accumulate toxins more quickly and in higher amounts than other species. Limited studies have shown that *A. irradians* is very sensitive to PSTs, leading to shell closure and reduced food intake [41].

PSTs induce oxidative stress, paralysis, and immune impairment of bivalves [40]. In this study, the two showed different physiological stress responses. As shown by KEGG pathway analysis, the bivalves presented both innate immune and inflammatory responses against PSTs exposure. However, there were differences in the pathways and genes between the two. The number of differential genes in *M. galloprovincialis* was two times higher than in *A. irradians*, which may be related to their pan-genome [42]. This implies that the large number of differential genes includes many dispensable genes in addition to the core genes. Due to the extreme intraspecific genetic diversity within *M. galloprovincialis*, it is expected that some of the differential genes were not related to the response to PSTs accumulation, but constitute background noise. However, it has also been proposed that accessory functions provided by the large number of dispensable genes may underpin the improved ability to adapt to challenging and varying environmental conditions [42]. The published data indicate that *M. galloprovincialis* displays adaptive traits to maintain immune capacity upon prolonged PSTs exposure, including a selective evolutionary advantage [43] and the enhancement of detoxification and antioxidant defense systems [44]. In contrast, the immune system of *A. irradians* displayed negative effects when faced with the same stress [45], as immuno-depleted, chronic [46], and uncontrolled inflammation caused persistent tissue damage [47]. In summary, accumulative metabolism and mortality reflect differences in external physiological characteristics, and *M. galloprovincialis* present stronger immune responses to PSTs exposure.

### 3.2. Interference-Specific Differences in Inflammation between M. galloprovincialis and A. irradians under PSTs Stress

Available data on bivalve immunity through the omics approach have only been obtained in the last few years. The inflammatory response is likely to be involved in repairing damaged tissues [48] and in detoxification [9]. These responses are time-, species-, and concentration-dependent [49]. Our results showed that there was a relatively complete inflammatory regulation system involving MAPK pathways in *M. galloprovincialis* but not in *A. irradians*. This is related to the stronger antioxidant capacity of mussels in the early stages [50]. In the Slow-stage, the extraordinary contribution of JNK/P38 revealed a dramatic inflammatory response in *M. galloprovincialis*. JNK activation is a key determinant in the cellular response. The activation of the JNK pathway was consistent with the metabolic rule of PSTs. Some researchers have suggested that JNK plays an active role in toxin stress [51].

In contrast, *A. irradians* displayed immunosuppression after exposure to shellfish toxins such as okadaic acid [52], domoic acid [53], and micropolystyrene [54]. In this study, the expression of inflammation-related genes of *A. irradians* showed non-significant and significant inhibition, which may indicate the immunosuppression of *A. irradians* against PSTs as well, but the immunosuppression indicators still need to be measured. Continuous upregulation of signal transduction activated the NF-κB1 in the Slow-stage of *M. galloprovincialis*, while this pathway was significantly down-regulated in the Fast-stage in *A. irradians*. Therefore, the two species are diametrically opposite in terms of cell survival and apoptosis regulation. Therefore, an unexpected finding was that the PI3K-AKT signaling pathway was significantly upregulated in the transcriptome of *A. irradians* at the Slow-stage. Additionally, the PI3K-AKT signaling pathway has previously been shown to have a synergistic effect with MAPK in promoting the inflammatory reaction [55]. To summarize, PST-induced metabolic disorders are likely to increase susceptibility to pathogens by physiologically weakening bivalves and interfering with immune functions for tissue repair [1].

### 3.3. Importance of Adjusting the Immune Balance between Inflammation and Anti-Inflammatory Effects

In model organisms such as shellfish, the inflammatory balance reaction [56,57] is one of the main pathways affecting metabolism and autophagy. In this study, factors such as PPP2R1A were significantly expressed in *M. galloprovincialis*, suggesting that AMPK could inhibit MAPK. The metabolic regulation process of mussels for toxins is more diversified. As a result of the self-limitation of the p38 MAPK signaling pathway and activation of the AMPK signaling pathway to inhibit the MAPK/NF-κB pathway, damage to the body of the mussel is significantly reduced through a dynamic balance. Studies have also confirmed the presence of stress-memory in *M. galloprovincialis* involving different responses at different times and eventual survival, such as from inflammation to control and resolve the inflammatory response and avoid subsequent cellular death and DNA damage after infection with *Vibrio splendidus*. The combination of a complex genome with the adaptation of the mussel immune system to a changing environment could explain this high variability [42,43]. The internal regulation of the MAPK pathway and the exogenous resistance regulation of AMPK can reflect the fatigue in the stress response of mussels. This includes the slowing of toxin metabolism and the adaptive down-regulation of the inflammatory response.

Novel insights into the specificity of AMPK signaling complexes have been gained [58]. Studies have confirmed the inhibitory effect of AMPK on inflammatory pathways such as JAK-STAT [59] and NF-κB [60]. The role of AMPK linked with various anti-inflammatory signals to limit the inflammatory response has been widely confirmed [61]. In *M. galloprovincialis*, this signaling pathway forms a perfect balance with MAPK to control damage to the body. In contrast, the stress response of MAPK was relatively weak in *A. irradians*, and its balance regulation was self-limited, not via AMPK. We believe that this inflammatory balance system was closely related to the dominant physiological characteristics and immune advantages of *M. galloprovincialis*. However, *A. irradians* experienced higher mortality without such balanced regulation. In contrast, this unbalanced regulation simultaneously enables cells to overcome metabolic stress by inhibiting AMPK signaling [61], a key singular node of cellular metabolism in *A. irradians*. AMPK and PI3K-AKT are involved in the regulation of metabolites such as glucose. Therefore, we believe that the detoxifying system of *A. irradians* is more inclined to cellular metabolizing and apoptosis than toward inflammation regulation and self-protection.

This is the first study to evaluate the differences in detoxification mechanisms for paralytic shellfish toxin in *M. galloprovincialis* and *A. irradians* under natural algal bloom conditions. In order to explain the stress response difference between the two shellfish in the process of toxin metabolism, we divided the process into two metabolic stages. However, due to the long time span of the sampling, we could not accurately determine the activation time of AMPK, and thus further detailed research is needed. *A. irradians* exhibited high mortality, and although we were able to ensure that the experiments were conducted in sufficient numbers, *A. irradians* were taken alive and did not accurately reflect the condition of the large number of dead scallops. We mainly used the KEGG database for data analysis, which is strongly biased towards model organisms. The vast majority of bivalve genes only show limited sequence homology with sequences with a known function, which can often lead to incorrect annotations, and the signaling pathways in bivalves are not necessarily identical to those found in other organisms. In addition, autophagy and tissue damage are also common features in shellfish exposed to PSTs, although no relevant experiments were performed in this study.

## 4. Conclusions

An effective immune response is indispensable for growth, reproduction, and survival. In this study, we comprehensively compared transcription and physiological responses to examine the inflammatory–anti-inflammatory balance mechanisms in *M. galloprovincialis* and *A. irradians* under PSTs exposure. The significant changes found involved MAPK and AMPK signaling pathways and the specific regulatory pathway, PI3K-AKT. JNK and p38 MAPK activate the MAPK pathway in *M. galloprovincialis*. Meanwhile, there was significant expression of inhibitor genes, including TNF, NF-κB, and AMPK, especially during the slow metabolic phase. Therefore, an intense inflammatory reaction and a corresponding anti-inflammatory reaction in *M. galloprovincialis* constituted a relatively effective mechanism for high-filtering activity and low-tissue damage after PSTs exposure. However, *A. irradians* focused on the regulation of downstream cell metabolism and apoptosis, and thus the immune imbalance resulted in serious tissue damage and high mortality. In summary, there were clear differences in the metabolic patterns related to PSTs in *M. galloprovincialis* and *A. irradians*. Inflammation is an important factor that affects the metabolic pattern of PSTs in bivalves.

## 5. Materials and Methods

### 5.1. Sample Collection and Preparation

The exposure experiment was conducted in the Qinhuangdao coastal culture area (39.94211° N, 119.73559° E). One-year-old *M. galloprovincialis* (shell length: 46 mm ± 3.8 mm) and *A. irradians* (shell length: 65 ± 4.0 mm) were cultured in shell breeding cages under surface seawater (depth: 10 m) from January 2019. An *Alexandrium catenella* red tide was observed from 29 March until 13 May (<200 cells/L), with the highest density at 3.40 × 10^3^ cells/L. From 2 April, sampling was performed every 3–6 days (depending on the sea conditions). Six biological replicates (PSTs accumulation × 3 and RNA-seq × 3) were collected each time, and each biological replicates contained 3–5 units. Three groups sampled on 11 April, 27 April, and 7 May were chosen for transcriptomics research. Shellfish hepatopancreas was collected on ice, washed with normal saline, and drained of surface water. All samples were immediately frozen in liquid nitrogen. Samples for sequencing were stored at −80 °C, whereas the samples for PSTs concentration measurements were stored at −20 °C until further analysis.

### 5.2. Determination of PSTs Accumulation

Five grams of each sample was weighed into a 50 mL centrifuge tube after mixing with a homogenizer (T18, IKA, Staufen, Germany), and 5 mL of 1% acetic acid aqueous solution was added for extraction. After vortexing for 10 min, the mixture was heated using a boiling water bath for 5 min and allowed to cool to room temperature in flowing water. The samples were centrifuged at 10,000× *g* for 5 min, and 1 mL of the supernatant was removed and adjusted for pH by 5 µL ammonium hydroxide, then centrifugation for 10 min at 16,000× *g* for purification of solid-phase extraction (SPE). ENVI-Carb cartridges (250 mg, 3 mL, Supelco, Bellefonte, PA, USA) were activated with 3 mL acetonitrile, 3 mL 20% acetonitrile aqueous solution (containing 1% acetic acid), and 3 mL 0.1% ammonium hydroxide. After activation, 500 µL of the supernatant was added, and 700 µL ultrapure water was used for elution. Finally, 1 mL 75% acetonitrile solution (containing 0.25% formic acid) was used for elucidation. All samples were filtered through 0.22 μm syringe filters.

### 5.3. LC-MS/MS Analysis

LC-MS/MS analysis of the PSTs was performed using an HPLC system (LC-20ADXR, Shimadzu, Kyoto, Japan) coupled to a hybrid triple quadrupole linear ion trap mass spectrometer (5500 QTRAP, AB Sciex, Boston, MA, USA). PSTs were screened using the multiple reaction monitoring (MRM) method for the separation of toxins using a hydrophilic column (TSK-Amide-80, 150 mm × 2.0 mm, 3 µm particle size) [62]. The PSTs standards, including C1 and 2, GTX2 and 3, GTX1 and 4, GTX5, and STX were purchased from the National Research Council of Canada. Thirteen PSTs standards diluted to 100 μg/mL were implemented as quality controls (QC) in the analytical runs (Tables S5–S7).

### 5.4. RNA Extraction, Library Construction, and Sequencing Analysis

The hepatopancreas of shellfish were used for RNA extraction. RNA extraction and transcriptomic sequencing were performed by Gene Denovo, Guangzhou. The mRNA was enriched by Oligo (dT) beads. Sequencing was performed on an Illumina HiSeqTM 4000 platform. The paired-end sequencing with a 2 × 150 bp approach was applied. A total of 18 libraries were established (including 2 species × 3 time points × 3 biological replicates). Clean data were produced by removing adaptor sequences, unknown nucleotides (>10%), and low-quality sequences (Q-value ≤ 10) from the raw data for subsequent analyses. Then, transcriptome de novo assembly was carried out with the short reads assembling program Trinity. For each transcript, an FPKM value was calculated to quantify its expression abundance and variation using RSEM software. The unigene expression was calculated and normalized to RPKM (Reads Per kb per Million reads). Raw sequencing data has been uploaded to NCBI and the relevant accession numbers are PRJNA850029 and PRJNA850516.

### 5.5. Differentially Expressed Genes (DEGs) Analysis

Differential expression analysis was performed using DESeq2 software. The read count was first normalized, and then the probability of hypothesis testing (*p*-value) was calculated according to the model. Finally, multiple hypothesis tests were performed to correct the FDR values. We conducted two-by-two comparisons for the sampling groups 11 April, 27 April, and 7 May, respectively, where we set 11 April to 27 April as the Fast-stage and 27 April to 7 May as the Slow-stage. Based on the results of analysis of variance, genes with *p*-value < 0.05 and |log_2_FC| > 1 were screened as significantly different genes. GOseq software was used to annotate the GO functions in order to reveal the biological functions of the differentially expressed genes; KEGG was used to analyze the signal pathways involving the genes. A threshold-corrected *p*-value < 0.05 was used to determine the significance of differences in GO and KEGG annotations of DEGs. Moreover, we performed gene set enrichment analysis using the software GSEA and MSigDB to identify whether a set of genes in a specific KEGG pathway showed significant differences between the two groups. Significantly enriched gene sets were defined as those with normalized enrichment score (NES) > 1 and *p*-value < 0.05.

### 5.6. Validation of Gene Expression by qRT-PCR

To validate the reliability of our transcriptome sequencing data, 12 DEGs of *M. galloprovincialis* and *A. irradians* were selected and analyzed by qRT-PCR. We selected eight common primers, TRAF3, MAP3K7, NF-κB1, CDC42, RAC1, PPP2R1A, CSNK2B, DUSP7, and four characteristic primers, JUN and GADD45B in *M. galloprovincialis* and BIRC2 and GADD45A in *A. irradians*. The primers and reference genes are listed in Supplementary Table S8.

The total reaction volume of 20 μL contained 4 μL 4× gDNA wiper Mix, 2 μL of total RNA, 4 μL of 5× HiScript‖qRT SuperMix‖, and 10 μL of RNase-Free ddH_2_O. The amplification profile was as follows: denaturation at 95 °C for 90 s followed by 40 cycles of 95 °C for 5 s, 60 °C for 15 s, and 72 °C for 20 s as described previously. The GAPDH gene was chosen as a reference gene. Relative mRNA expression was determined using the 2^−ΔΔCT^ method.

### 5.7. Statistical Analysis

All data were analyzed using SPSS version 16.0 for Windows (SPSS, Chicago, IL, USA). Two-tailed Student’s *t*-tests were used to analyze differences among treatments. The level of significance was set at *p* < 0.05. All data were presented as mean ± standard deviation (SD).

## Figures and Tables

**Table 1:** GSEA analysis parameters of the target pathways.

	*M. galloprovincialis*				*A. irridians*			
	Fast-Stage		Slow-Stage		Fast-Stage		Slow-Stage	
	NES	*p*-Value	NES	*p*-Value	NES	*p*-Value	NES	*p*-Value
MAPK	1.13	-	1.82	***	1.06	-	−1.31	*
TNF	0.88	-	2.01	***	1.24	*	−1.28	*
Ras	1.04	-	1.50	***	1.01	-	−0.87	-
NF-κB	0.82	-	1.74	***	1.38	*	0.85	-
AMPK	−1.55	***	1.85	***	−1.27	-	−1.15	-
PI3K-Akt	-	-	-	-	1.12	-	1.11	-

Notes: Normalized enrichment score (NES) > 1 and *p*-value < 0.05 were considered statistically significant (* *p* < 0.05, ** *p* < 0.01, *** *p* < 0.001).

## Data Availability

RNA-seq raw sequencing data has been uploaded to NCBI’s Sequence Read Archive (SRA) and the relevant accession numbers are PRJNA850029 and PRJNA850516.

## Supplementary Materials

The following supporting information can be downloaded at: https://www.mdpi.com/article/10.3390/toxins14080516/s1, Table S1: Distribution dynamics of *Alexandrium catenella* cells; Table S2: Summary of transcriptome sequencing; Table S3: Summary of reads annotation; Table S4: KEGG enrichment results of TOP30; Table S5: Results of toxin concentration (μg STX eq/kg); Table S6: Concentration of mixed standard solution; Table S7: Results of quality control; Table S8: PCR primer list.
