# Supplementary material for: Transcriptome Analysis Reveals MAPK/AMPK as a Key Regulator of the Inflammatory Response in PST Detoxification in Mytilus galloprovincialis and Argopecten irradians
Source: Toxins (Basel). 2022 Jul 28;14(8):516. doi: 10.3390/toxins14080516 (PMC9416634; doi:10.3390/toxins14080516)
Supplement: Supplementary file 1 [file toxins-14-00516-s001.zip › toxins-1805520-supplementary.pdf]

toxins

MDPI

Supplementary Materials: Transcriptome Analysis Reveals MAPK/AMPK as a Key Regulator of the Inflammatory Response in PST Detoxification in *Mytilus galloprovincialis* and *Argopecten irradians*

Chenfan Dong, Haiyan Wu, Guanchao Zheng, Jixing Peng, Mengmeng Guo and Zhijun Tan

Table S1. Distribution dynamics of *Alexandrium catenella* cells.

Date	3.20	3.29	4.2	4.8	4.11	4.16	4.19	4.23	4.27	5.2	5.7	5.13
Abundances Cells·L^{-1}	193 ±12	560 ±53	1000 ±100	2446 ±150	2910 ±130	3396 ±155	2656 ±150	2043 ±235	1163 ±152	743 ±121	493 ±101	223 ±40

Table S2. Summary of transcriptome sequencing.

Species	Libraries	Raw reads	Clean reads	Average reads length (bp)	Q30 (%)	GC (%)
M. galloprovincialis	1	73063150	72998660	150	93.69%	44.26%
	2	54171196	54109470	150	93.39%	44.26%
	3	58196756	58142204	150	93.63%	43.34%
	4	53820646	53776456	150	93.82%	43.60%
	5	56362972	56313774	150	94.28%	43.68%
	6	47209170	47164074	150	93.68%	45.12%
	7	58189742	58146404	150	94.14%	47.04%
	8	62910802	62849256	150	93.19%	45.23%
	9	64824906	64753942	150	93.03%	45.24%
A. irradians	1	50033282	49974964	150	94.2%	46.58%
	2	51502068	51432052	150	93.52%	46.56%
	3	46783174	46713534	150	94.02%	45.96%
	4	52632854	52556582	150	93.38%	45.60%
	5	50350636	50286476	150	94.11%	47.92%
	6	54297322	54219582	150	93.03%	47.08%
	7	60582164	60492530	150	93.39%	45.74%
	8	42911322	42870610	150	94.27%	47.79%
	9	51756686	51673074	150	92.81%	45.42%

Table S3. Summary of reads annotation.

	M. galloprovincialis	*A. irridians*
Total Unigenes	100863	70011
Nr	45015	28827
Swissport	22297	15949
KOG	18040	13294
Kegg	20795	14761

Annotation gene	45220	29572
Without annotation gene number	55643	40439

Table S4. KEGG enrichment results of TOP30.

	Pathway ID	Pathway name	Number of DEGs
A. irradians	KO04151	PI3K-Akt signaling pathway	357
	KO04210	Apoptosis	344
	KO04145	Phagosome	332
	KO04921	Oxytocin signaling pathway	319
	KO04142	Lysosome	314
	KO04020	Calcium signaling pathway	308
	KO04022	cGMP - PKG signaling pathway	240
	KO04024	cAMP signaling pathway	240
	KO04010	MAPK signaling pathway	234
	KO04120	Ubiquitin mediated proteolysis	204
	KO04371	Apelin signaling pathway	194
	KO04070	Phosphatidylinositol signaling system	175
	KO04512	ECM-receptor interaction	166
	KO04750	Inflammatory mediator regulation of TRP channels	166
	KO04625	C-type lectin receptor signaling pathway	163
	KO04146	Peroxisome	151
	KO00480	Glutathione metabolism	142
	KO04612	Antigen processing and presentation	130
	KO04150	mTOR signaling pathway	129
	KO04066	HIF-1 signaling pathway	128
	KO04668	TNF signaling pathway	128
	KO04624	Toll and Imd signaling pathway	115
	KO04152	AMPK signaling pathway	106
	KO04330	Notch signaling pathway	104
	KO03320	PPAR signaling pathway	101
	KO00980	Metabolism of xenobiotics by cytochrome P450	88
	KO04657	IL-17 signaling pathway	84
	KO04666	Fc gamma R-mediated phagocytosis	80
	KO04115	p53 signaling pathway	56
	KO04630	Jak-STAT signaling pathway	38
M. galloprovincialis	KO04015	Rap1 signaling pathway	401
	KO04921	Oxytocin signaling pathway	389
	KO04014	Ras signaling pathway	341
	KO04010	MAPK signaling pathway	332
	KO04020	Calcium signaling pathway	313
	KO04022	cGMP - PKG signaling pathway	310
	KO04621	NOD-like receptor signaling pathway	304

KO04024	cAMP signaling pathway	290
KO04512	ECM-receptor interaction	285
KO04064	NF-kappa B signaling pathway	245
KO00480	Glutathione metabolism	228
KO04625	C-type lectin receptor signaling pathway	214
KO04371	Apelin signaling pathway	213
KO04668	TNF signaling pathway	213
KO04670	Leukocyte transendothelial migration	210
KO04612	Antigen processing and presentation	207
KO04912	GnRH signaling pathway	197
KO04066	HIF-1 signaling pathway	189
KO04150	mTOR signaling pathway	179
KO04330	Notch signaling pathway	175
KO04624	Toll and Imd signaling pathway	174
KO04310	Wnt signaling pathway	173
KO04657	IL-17 signaling pathway	140
KO00980	Metabolism of xenobiotics by cytochrome P450	139
KO03320	PPAR signaling pathway	139
KO04152	AMPK signaling pathway	138
KO04115	p53 signaling pathway	125
KO04666	Fc gamma R-mediated phagocytosis	107
KO04660	T cell receptor signaling pathway	89
KO04630	Jak-STAT signaling pathway	48

Table S5. Results of toxin concentration (μg STX eq/kg).

M. galloprovincialis									
Date	STX （TEF=1）	GTX1 （TEF=1）	GTX2 （TEF=0.4）	GTX3 （TEF=0.6）	GTX4 （TEF=0.7）	GTX5 (TEF=0.1)	C1 (TEF=0.1)	C2 （TEF=0.1）	Content
4.2	0.000	582.570	0.000	19.342	426.978	0.000	0.000	0.000	893.060
4.8	140.196	2778.853	144.081	109.407	1276.060	0.000	6.984	6.228	3936.889
4.11	156.477	2965.478	61.416	61.451	1579.437	0.000	9.674	8.651	4290.830
4.16	0.000	3938.977	250.910	623.582	1701.856	0.000	0.000	0.000	5604.789
4.19	73.136	1295.834	328.317	771.545	681.590	0.000	0.000	0.000	2440.337
4.23	71.782	668.912	471.328	1057.776	293.802	0.000	0.000	0.000	1769.553
4.27	37.922	826.867	16.406	31.609	425.387	0.000	0.000	0.000	1188.088
5.02	53.246	445.110	68.311	152.506	202.212	0.000	0.000	0.000	758.732
5.07	0.000	365.509	40.007	81.554	154.325	0.000	0.000	0.000	538.471
5.13	80.923	118.497	64.192	24.194	30.683	0.000	0.000	0.000	261.091
5.27	0.000	10.707	40.622	40.586	11.459	0.000	0.000	0.000	59.328
A. irradians									
Date	STX （TEF=1）	GTX1 （TEF=1）	GTX2 （TEF=0.4）	GTX3 （TEF=0.6）	GTX4 （TEF=0.7）	GTX5 (TEF=0.1)	C1 (TEF=0.1)	C2 （TEF=0.1）	Content

4.2	8.370	33.098	4.239	6.801	43.022	3.933	8.130	5.297	79.096
4.8	15.049	107.541	15.509	12.981	143.041	0.000	3.838	1.937	237.289
4.11	35.940	354.415	31.431	20.382	251.024	0.000	16.800	6.647	593.218
4.16	60.984	0.000	215.468	1472.011	0.000	0.000	0.000	0.000	1030.378
4.19	46.740	37.510	185.444	900.865	27.060	27.054	0.000	0.000	720.593
4.23	29.906	0.000	95.975	999.512	13.012	0.000	0.000	0.000	677.112
4.27	88.554	426.187	51.111	34.590	125.941	0.000	0.000	0.000	644.098
5.02	29.200	0.000	111.627	778.961	17.560	21.614	0.000	0.000	555.681
5.07	12.217	227.523	53.303	266.214	140.147	0.000	0.000	0.000	518.892
5.13	78.150	102.302	149.013	207.479	67.411	0.000	35.567	12.333	416.523
5.27	66.559	0.000	24.258	21.656	0.000	0.000	0.000	0.000	89.256

Table S6. Concentration of mixed standard solution.

	500PPB	200PPB	100PPB	50PPB	20PPB	10PPB
C1&2-b	669.03	267.61	133.81	66.90	26.76	13.38
	200.00	80.00	40.00	20.00	8.00	4.00
GTX1&4-d	635.56	254.22	127.11	63.56	25.42	12.71
	200.00	80.00	40.00	20.00	8.00	4.00
GTX2&3-d	471.72	188.69	94.34	47.17	18.87	9.43
	200.00	80.00	40.00	20.00	8.00	4.00
GTX5-c	500.00	200.00	100.00	50.00	20.00	10.00
STX-f	500.00	200.00	100.00	50.00	20.00	10.00

Table S7. Results of quality control.

STX

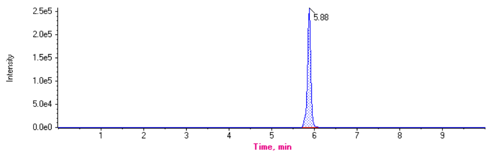

GTX1

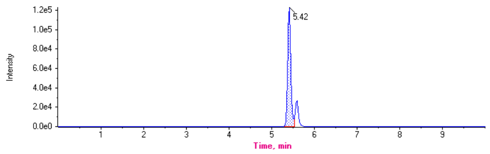

GTX2

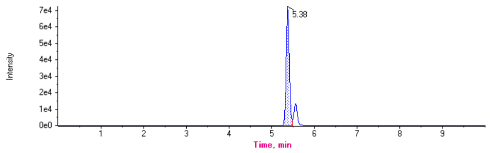

GTX3

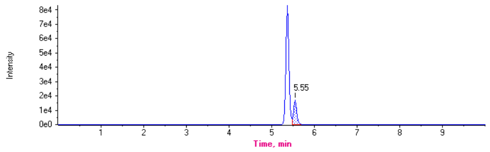

GTX4	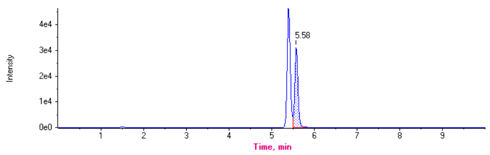	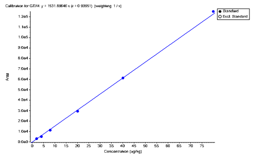
GTX5	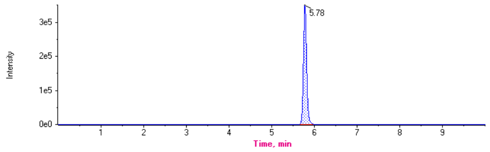	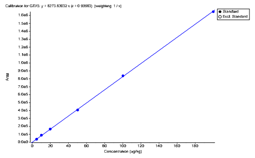
C1	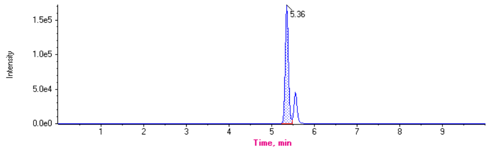	
C2	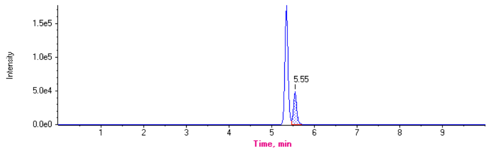	

Table S8. PCR primer list.

	A. irridians			*M. galloprovincialis*		
Gene symbol		5'--3'	Tm		5'--3'	Tm
Traf3	Ai-traf3-Fw	CTTGGAACCCCCGACGATAC	61.6	Me-traf3-Fw	AGCCCCACCTTCTACATCCT	57.6
	Ai-traf3-Rv	GAACATCCTTCAGACGCCCT	58.9	Me-traf3-Rv	TCCTGCTGTCCCACAATCAC	58.6
MAP3K7	Ai-map3k7-Fw	GCGGGGTTGTATCCTGAGAG	59.4	Me-map3k7-Fw	GGGGGTAAGGTGTTATTGGCT	59.9
	Ai-map3k7-Rv	CACCTTCCTTAGCAGCGAGT	57.2	Me-map3k7-Rv	AACAGTAGTAGTGGCAGCAGT	51.8
Birc2	Ai-birc2-Fw	TACACTGGAAGGTGGGGATG	57.8	-	-	-
	Ai-birc2-Rv	GGCTGAGACTGGCGATAGAG	57.3	-	-	-
NF-κB1	Ai-nfkb1-Fw	GACCCTATCTGCGTTGCTGG	60.5	Me-nfkb1-Fw	GCTGCCAAATGTAAGGGTGTC	59.7
	Ai-nfkb1-Rv	TTCTGTCGCCTGGAAAACCT	59.7	Me-nfkb1-Rv	TGCGAAAAAGCCTCCAATGT	60.7
CDC42	Ai-cdc42-Fw	ACTGCGGAACTCTTCAACCC	59	Me-cdc42-Fw	CGGCTTCAAAACAGCAGAGT	58
	Ai-cdc42-Rv	CATTCATCTGGCTTCGGGCA	64	Me-cdc42-Rv	GCAAGAGAACTAAGGGCGGT	58.7
RAC1	Ai-rac1-Fw	CCAGCCACACTGCTACTTCA	56.8	Me-rac1-Fw	CCAGTCCCTGTGGGTATGT	54.7
	Ai-rac1-Rv	GCCCGAGTTTCACGACCATT	62.1	Me-rac1-Rv	GGTTGGTGATGGTGCTGTTG	59
PPP2R1A	Ai-ppp2r1a-Fw	CATCTGTTGCCCCTGTTCCT	59.6	Me-ppp2r1a-Fw	CTGACGACTCTCTCTACCCAA	54.3
	Ai-ppp2r1a-Rv	GTTCAATGTCGCTGCCTCAC	58.6	Me-ppp2r1a-Rv	ACGCCAAGTGCCAATGCT	59.5
CSNK2B	Ai-csnk2b-Fw	CACCCCTAAGTCATCTCGCC	59.4	Me-csnk2b-Fw	TTGGTTTTGTGGATTGAGAGG	57.6
	Ai-csnk2b-Rv	GCAGGTCGTTTTGGTCTGTATTC	60.6	Me-csnk2b-Rv	CCAAAATAGGCTCCATCAGTGT	58.6
GADD45A	Ai-gadd45a-Fw	GGATGACTGGGCGTGGATAA	60.4	-	-	-
	Ai-gadd45a-Rv	CCTACTGCTGGGAGAACGAC	57.1	-	-	-
DUSP7	Ai-dusp7-Fw	ACAGTTGGCGGTTTTGATTGG	62.3	Me-dusp7-Fw	ACGAGACTTATGCGAAGGGAC	58.6
	Ai-dusp7-Rv	CTGTCTGGCGGGCATTAGTC	60.5	Me-dusp7-Rv	TGGGTATTGTGAGCGAGGTG	59
GAPDH	Ai-gapdh-Fw	AACGGTCTTCTGTGTAGCGG	57.7	Me-gapdh-Fw	ACAGTTGAAGCAAAGGATGGA	57.3
	Ai-gapdh-Rv	ATTCCTTGGGGTTCTGCTGG	61.3	Me-gapdh-Rv	GGTTGTGAAGACACCAGTAGA	52.1
JUN	-	-	-	Me-jun-Fw	TGAACACATAGTCAAGGCACAGA	58.3
	-	-	-	Me-jun-Rv	ACGACAGGATGAAGAACCACA	57.5

GADD45B	-	-	-	Me-gadd45b-Fw	TGCTGTCTGTCTTAGCGTTCT	55.3
	-	-	-	Me-gadd45b-Rv	GGCACAGGAAGATGGCAGA	58.8
